# Ball-milled zinc nickel sulfide nanostructures on nickel substrate for enhanced electrochemical methanol sensing

**DOI:** 10.1039/d6ra02062c

**Published:** 2026-06-05

**Authors:** N. Roushdy, Mervet Ramadan, Samah. M. Yousef, A. A. M. Farag, Amany S. El-Khouly, M. Y. Nassar, Rasha Abu-Khudir, M. S. Ayoup, Mohamed S. Elnouby, Elbadawy A. Kamoun

**Affiliations:** a Electronics Materials Department, Advanced Technology and New Materials Research Institute, City of Scientific Research and Technological Applications (SRTA-City) New Borg El-Arab City P.O. Box 21934 Alexandria Egypt; b Physics Department, Faculty of Science, Alexandria University Alexandria Egypt; c Basic Science Department, Higher Institute of Engineering and Technology New Borg El-Arab City Alexandria Egypt; d Thin Film Laboratory, Physics Department, Faculty of Education, Ain Shams University Heliopolis, Roxy Cairo 11757 Egypt; e Department of Chemistry, College of Science, King Faisal University Al-Ahsa 31982 Saudi Arabia ekamoun@kfu.edu.sa mynassar@kfu.edu.sa; f Nanomaterials and Composites Research Department, Advanced Technology and New Materials Research Institute, City of Scientific Research and Technological Applications (SRTA-City) New Borg El-Arab City Alexandria 21934 Egypt M_nano2050@yahoo.com

## Abstract

Zinc-nickel sulfide (ZnNiS) nanostructures were synthesized *via* a simple ball-milling process followed by low-temperature annealing and investigated exclusively as a surface-modifying layer for electrochemical methanol sensing on conductive substrates. Structural characterization using X-ray diffraction, Fourier-transform infrared spectroscopy, scanning and transmission electron microscopy confirmed the formation of nanocrystals with a mixed orthorhombic–trigonal phase and particle sizes in the range of 50–150 nm. BET analysis revealed a mesoporous morphology with a high specific surface area, while thermal analysis confirmed stability up to 550 °C, supporting their suitability for electrochemical applications. Electrochemical measurements using cyclic voltammetry and linear sweep voltammetry demonstrated that methanol oxidation is primarily driven by the nickel substrate, with ZnNiS acting as a nanostructured catalytic modifier that enhances active surface sites and facilitates charge transfer processes. The optimized electrode configuration exhibited sensitivities of 62.785 µA mM^−1^ on Ni-based electrodes and 3.8214 µA mM^−1^ on stainless steel, confirming the dominant role of the substrate in governing the overall response. Kinetic analysis indicated pseudo-second-order adsorption behavior, consistent with chemisorption-controlled electrooxidation of methanol. Overall, the study highlights the synergistic interaction between ZnNiS nanostructures and the Ni substrate, leading to improved electrochemical sensing performance for cost-effective methanol detection.

## Abstract 2

Zinc-nickel sulfide nanostructures were synthesized *via* a simple ball-milling process followed by low-temperature annealing and investigated exclusively as a surface-modifying layer for electrochemical methanol sensing on conductive substrates.

## Graphical abstract

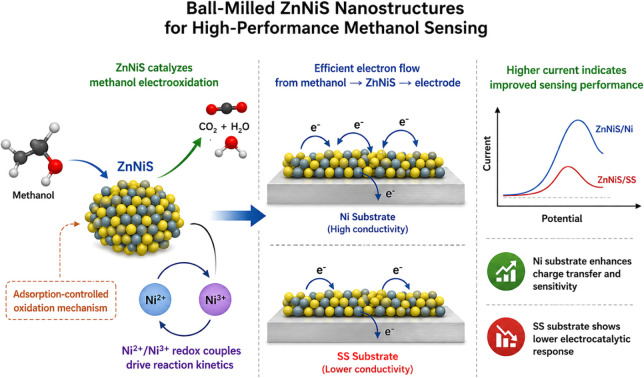

## 1 Introduction

Semiconductor nanoparticles exhibit unique physicochemical properties, which have driven extensive research interest over the past decade.^1^ Among these materials, metal chalcogenide nanostructures, particularly transition metal sulfides, have emerged as highly efficient platforms for electrochemical sensing and electrocatalytic oxidation reactions due to their tunable electronic structures and fast interfacial charge-transfer characteristics.^2–4^ These intrinsic properties enable strong electrocatalytic responses, making them suitable for sensitive and stable detection systems.^5^ In electrochemical sensing, the overall performance is governed primarily by electron-transfer kinetics, adsorption–desorption processes, and catalytic redox activity, which determine sensitivity, selectivity, and stability.^6,7^

A broad range of semiconductor materials, including metal oxides and metal sulfides, has been investigated for sensing applications. Although metal oxides often show good selectivity, their limited long-term electrochemical stability has shifted research attention toward metal sulfides.^2^ Among sulfide-based systems, ZnS is attractive due to its chemical stability and tunable electronic structure, while Ni-based sulfides are well known for their high electrocatalytic activity toward methanol electrooxidation driven by the reversible Ni^2+^/Ni^3+^ redox couple.^13–15^ The selection of ZnNiS in this work is therefore rationalized by combining the structural stability of ZnS with the intrinsic methanol oxidation activity of Ni-based sulfides, enabling enhanced charge-transfer kinetics and improved catalytic efficiency. Furthermore, II–VI semiconductors such as ZnS, CdS, and ZnO offer versatile electronic configurations that support redox-mediated processes relevant to electrochemical sensing and energy applications.^4^

Electrochemical techniques, including cyclic voltammetry (CV) and electrochemical impedance spectroscopy (EIS), are widely used to elucidate charge-transfer mechanisms and reaction kinetics at modified electrode interfaces.^9^ Methanol electrooxidation has attracted considerable attention due to its importance in fuel cells, industrial processing, and environmental monitoring.^10–12^ Transition-metal sulfides, including NiS, ZnS, and their mixed-metal derivatives, exhibit promising catalytic activity toward small organic molecule oxidation; however, achieving high efficiency requires rational control of electronic structure and interfacial charge transport rather than morphological enhancement alone.

Nickel-based sulfide systems play a central role in methanol electrooxidation because the Ni^2+^/Ni^3+^ redox couple facilitates electron transfer and accelerates oxidation kinetics at the electrode surface.^13–15^ This electrochemical mechanism provides the fundamental basis for the material design strategy adopted in this study.

The present study introduces ZnNiS nanostructures as a rationally engineered electrochemical sensing platform. The novelty of this work lies in the synergistic integration of Zn-induced structural stabilization with Ni-driven redox catalytic activity, which collectively enhances interfacial charge-transfer efficiency and methanol oxidation performance.^9,13–15^ Unlike conventional approaches that emphasize only surface morphology, this study highlights composition-driven electronic coupling and deposition-controlled structural evolution as the dominant factors governing electrochemical response. Systematic evaluation of deposition scan rate effects, reduction in charge-transfer resistance upon Zn incorporation, and stable catalytic behavior across multiple electrolytes provide a comprehensive understanding of the underlying electrocatalytic mechanism. Through integrated structural, morphological, and electrochemical analyses, the study establishes a clear correlation between compositionally engineered sulfide nanostructures and enhanced sensing performance.

This work aims to design, synthesize, and characterize high-performance Zn–Ni–S nanostructures and evaluate their electrocatalytic behavior for methanol oxidation. Specifically, the objectives are to (i) elucidate structural, morphological, and compositional properties using XRD, TEM, and EDS; (ii) investigate the influence of deposition scan rate on nanostructure evolution; and (iii) assess electrocatalytic performance *via* CV, LSV, and EIS to understand charge-transfer kinetics and methanol oxidation mechanisms.^1–15^ Ultimately, this study establishes ZnNiS nanostructures as a cost-effective and environmentally sustainable platform for advanced electrochemical sensing applications.

## 2 Experimental details

### 2.1 Synthesis of ZnNiS nanostructures

#### 2.1.1 Fabrication of ZnNiS nanoparticles

Zinc-nickel sulfide (ZnNiS) nanoparticles were synthesized *via* a controlled ball-milling assisted solid-state route, in which precursor chemistry and thermal activation collectively govern nucleation and phase evolution. Analytical-grade zinc acetate dihydrate (Zn(CH_3_COO)_2_·2H_2_O), nickel acetate tetrahydrate (Ni(CH_3_COO)_2_·4H_2_O), and thiourea (CH_4_N_2_S) were used as metal and sulfur sources without further purification. Under high-energy ball milling at 800 rpm for 20 min (ball-to-powder ratio 10 : 1), mechanical activation promotes intimate mixing and defect generation, facilitating enhanced reactivity between metal acetates and thiourea. During subsequent thermal treatment at 200 °C for 2 h, thiourea undergoes decomposition to release reactive sulfur species such as H_2_S and CS_2_, which react *in situ* with Zn^2+^ and Ni^2+^ ions to initiate nucleation of ZnS and NiS primary species. The initial stage of growth is therefore governed by heterogeneous nucleation of binary sulfide clusters, which subsequently interact through solid-state diffusion and interfacial bonding. As annealing proceeds, these intermediate ZnS and NiS nuclei undergo coalescence and compositional interdiffusion, leading to the formation of a thermodynamically stabilized mixed Zn–Ni–S phase. The final ZnNiS phase is formed through a diffusion-controlled solid-state transformation mechanism driven by cation exchange and lattice rearrangement. A secondary ball-milling step at identical conditions (800 rpm, 20 min, 10 : 1 ball-to-powder ratio) is then applied to eliminate soft agglomerates, refine crystallite domains, and enhance phase homogeneity without altering the established crystal structure. The overall process results in well-crystallized ZnNiS nanostructures with controlled phase evolution, demonstrating a reproducible solid-state reaction pathway suitable for electrochemical applications.

#### 2.1.2 Electrode fabrication and ink preparation

The synthesized ZnNiS nanoparticles were processed into a conductive ink for electrode fabrication with clearly defined composition and deposition parameters to ensure reproducibility. A total mass of 20 mg ZnNiS was used as the active material, combined with 10 mg polyvinylidene fluoride (PVDF) as the polymeric binder and 10 mg graphite as the conductive additive to enhance electron transport within the film. These components were dispersed in 2 mL of *N*,*N*-dimethylformamide (DMF) and ultrasonicated for 15 min at room temperature to obtain a homogeneous and stable suspension. The resulting ink was drop-cast onto pre-cleaned stainless steel and nickel substrates with a defined geometric active area of 1 cm^2^ (10 × 10 mm). The coated electrodes were then dried at 65 °C overnight to ensure complete solvent evaporation and formation of a mechanically stable and adherent composite film. The average coating mass on each electrode was controlled to maintain consistent loading across all samples, and the resulting film thickness was kept uniform through identical deposition conditions. This standardized fabrication protocol ensures reproducible electrode architecture, stable adhesion of the active layer, and reliable electrochemical performance across all measurements.

### 2.2 Structural, morphological, and compositional characterization

The crystal structure of the ZnNiS nanostructures was investigated using X-ray diffraction (XRD) on a Shimadzu 7000 diffractometer (CuK_α_ radiation, *λ* = 0.15406 nm) at 30 kV and 30 mA. Patterns were collected over a 2*θ* range of 10–60° with a scan rate of 2° min^−1^. Rietveld refinement was performed using EXPO-2014 software to extract precise lattice parameters, crystallite size, and phase composition.

Transmission electron microscopy (TEM, JEOL JEM2100 plus, Japan) provided detailed information on particle morphology, crystallinity, and size distribution, while scanning electron microscopy (SEM, JEOL JSM-6360LA, Japan) was employed to examine surface morphology and homogeneity. Before SEM imaging, samples were sputter-coated with a thin gold layer to prevent charging and enhance resolution.

Chemical bonding and molecular structure were analyzed using Fourier-transform infrared spectroscopy (FTIR, Bruker ALPHA, Germany). Raman spectroscopy (Horiba LabRam HR) was utilized to probe vibrational modes and confirm the structural integrity of the sulfide lattice, providing complementary information on atomic bonding and symmetry.

### 2.3 Electrochemical measurements

Electrochemical measurements were carried out using a Metrohm Autolab potentiostat (Model 87 070) under a standard three-electrode configuration. The working electrodes included bare nickel (Ni), bare ZnNiS-modified electrodes, and ZnNiS-coated nickel (ZnNiS/Ni) electrodes to systematically evaluate the individual and combined contributions of each component. A platinum rod was used as the counter electrode, and a saturated calomel electrode (SCE) served as the reference electrode. All measurements were performed in an aqueous electrolyte containing 1% NaCl with varying methanol concentrations.

Cyclic voltammetry (CV) and linear sweep voltammetry (LSV) were employed to investigate methanol oxidation behavior, electrocatalytic activity, and charge-transfer kinetics. The bare Ni electrode was used to evaluate the intrinsic activity of the substrate associated with the Ni^2+^/Ni^3+^ redox couple, while the bare ZnNiS electrode provided insight into the independent catalytic contribution of the nanostructured sulfide. The ZnNiS/Ni composite electrode was used to assess the synergistic interaction between ZnNiS and the Ni substrate. This systematic comparison enables clear separation of individual contributions and confirms that the enhanced performance arises from the combined effect of Ni-based redox activity and ZnNiS-mediated charge-transfer facilitation.

The analytical sensitivity of the electrodes was further assessed through the determination of the limit of detection (LOD) and limit of quantification (LOQ). These parameters were calculated based on the standard deviation of the blank response and the slope of the calibration curve obtained from the current-concentration relationship. The LOD and LOQ were estimated using signal-to-noise ratios of 3 and 10, respectively. The baseline noise was determined from repeated measurements of the supporting electrolyte under identical experimental conditions, ensuring the reliability and reproducibility of the calculated values.

### 2.4 Kinetic modeling of sensing performance

The sensing behavior was analyzed using pseudo-first-order and pseudo-second-order kinetic models, which are widely applied to describe adsorption-controlled electrochemical processes.

The pseudo-first-order kinetic model is expressed as:^16^1
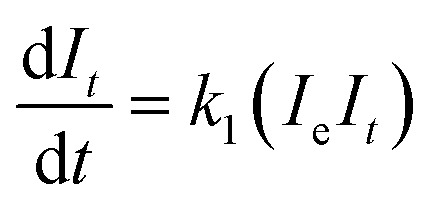


which, upon integration, yields the linearized form:

2 ln (*I*_e_ − *I*_*t*_) = ln *I*_e_ − *k*_1_*t*

The pseudo-second-order kinetic model is given by:3
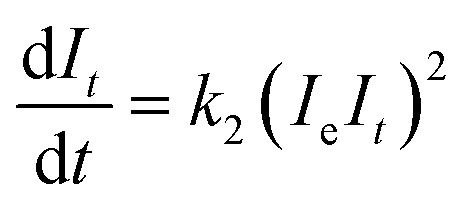


which can be rearranged into the following linear form:4
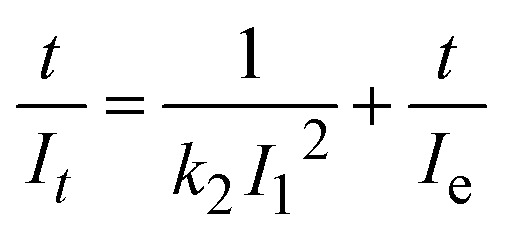
In these expressions, *I*_*t*_(A) represents the sensing current at time *t*, and *I*_e_(A) denotes the equilibrium current corresponding to the steady-state response at a given analyte concentration. The parameters *k*_1_(s^−1^) and *k*_2_(A^−1^ s^−1^) are the pseudo-first-order and pseudo-second-order rate constants, respectively.

The kinetic parameters were determined from the slopes and intercepts of the respective linear plots. The pseudo-first-order model is typically associated with diffusion-controlled processes, whereas the pseudo-second-order model reflects chemisorption involving electron sharing or transfer between the analyte and the active sites of the sensing material. The better-fitting model provides insight into the dominant sensing mechanism governing the electrochemical response.

### 2.5 Operational robustness, stability, and selectivity evaluation

To ensure the analytical reliability and practical applicability of the developed sensor, a comprehensive validation protocol was implemented, incorporating selectivity, interference resistance, reproducibility, stability, and limit of detection evaluation. Repeatability was assessed through multiple successive measurements using the same electrode, yielding consistent current responses with low relative standard deviation, confirming stable signal generation. Reproducibility was further validated using independently fabricated ZnNiS/Ni electrodes, which exhibited closely comparable electrochemical responses, demonstrating excellent fabrication consistency. Long-term stability was evaluated over an extended storage period, during which the sensor retained a high percentage of its initial current response, indicating strong structural integrity and electrochemical durability of the ZnNiS layer. Selectivity and interference studies were systematically conducted by introducing common coexisting species such as ethanol, glucose, and uric acid under identical conditions. The sensor response toward methanol remained significantly higher than that of interfering species, confirming high specificity and minimal cross-sensitivity. Furthermore, the limit of detection was calculated using the standard 3*σ*/*m* criterion, where *σ* represents the standard deviation of the blank signal and *m* is the slope of the calibration curve, ensuring statistically reliable quantification. These combined results establish that the proposed sensor exhibits robust analytical performance, high selectivity, excellent reproducibility, and reliable detection capability, making it suitable for practical electrochemical sensing applications.

## 3 Results and discussion

### 3.1 Morphological architecture and surface characteristics of ZnNiS nanostructures

**Fig. 1:**
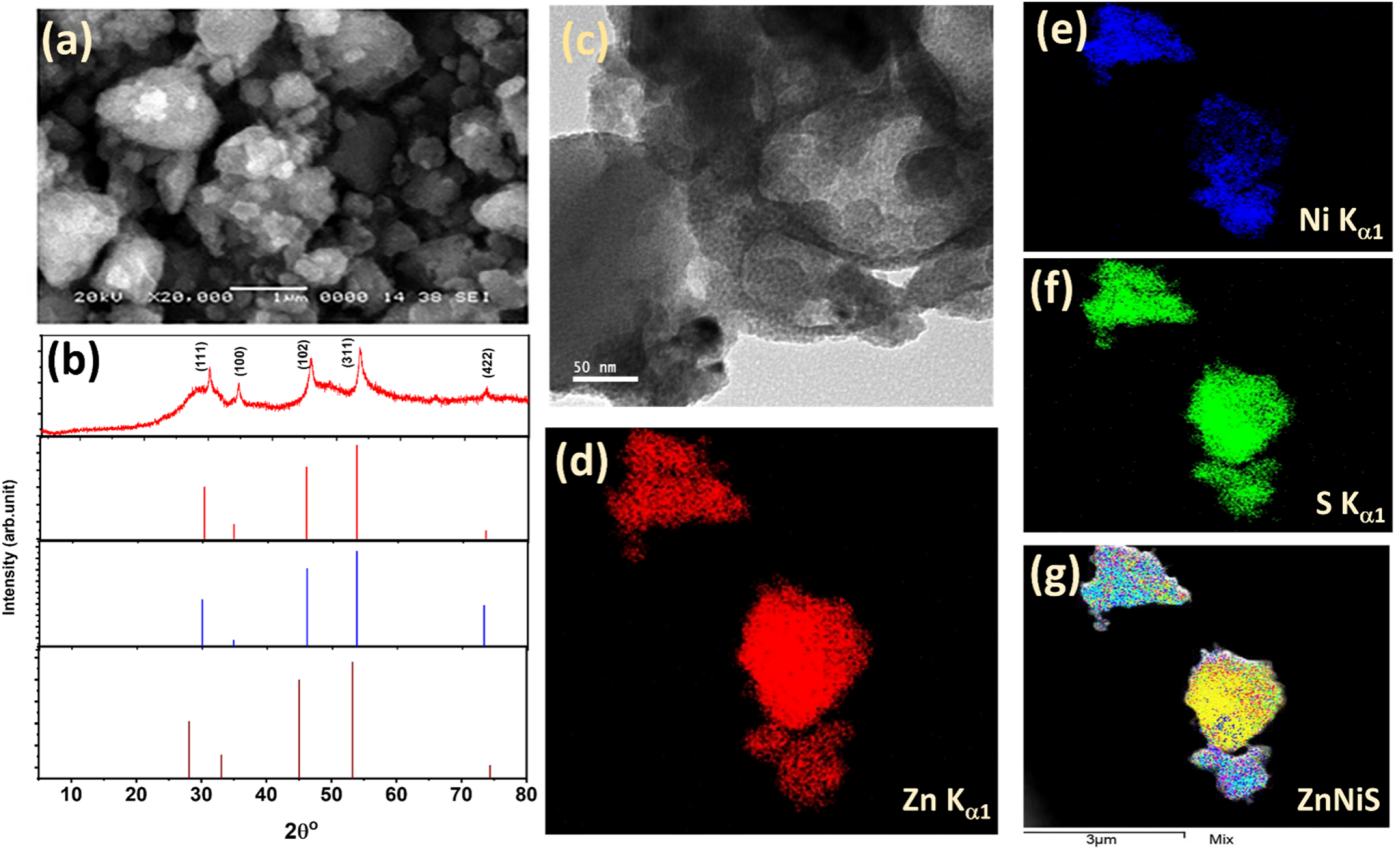
(a) SEM micrographs of ZnNiS nanoparticles showing particle morphology, (b) XRD pattern with structural refinement compared with reference patterns of ZnNiS_5_ and Zn(NiS_2_)_4_ phases, (c) HRTEM images revealing lattice features of the nanoparticles, and (d–g) elemental mapping illustrating the spatial distribution of Zn, Ni, and S.

The surface topography and spatial distribution of the synthesized ZnNiS nanoparticles were elucidated through scanning electron microscopy (SEM) at varying magnifications, as presented in Fig. 1(a). The micrographs reveal a densely packed assembly of granular nanostructures exhibiting a predominantly spherical to slightly irregular morphology. At lower magnification, the nanoparticles demonstrate remarkable homogeneity in coverage, forming a continuous network with minimal substrate exposure, a feature indicative of efficient nucleation and growth during the synthetic process. Upon closer examination at 200 00× magnification, individual particles become discernible, with dimensions ranging from approximately 50 to 150 nm.

The observed agglomeration represents an intrinsic characteristic of nanoscale materials rather than a synthetic shortcoming, arising from the high surface energy that drives interparticle attraction during post-synthesis processing.^17,18^ This phenomenon has been widely documented in ternary metal sulfides, where van der Waals forces and capillary effects during solvent evaporation promote the formation of clustered architectures.^19,20^ Notably, the degree of aggregation observed herein is considerably less pronounced than that reported for analogous ZnS–NiS composites synthesized *via* conventional precipitation methods, where particle sizes frequently exceed 500 nm with severe coalescence.^21^ The relatively controlled aggregation in our system suggests that the adopted synthetic protocol effectively moderates surface energy through either capping agent interactions or controlled reaction kinetics.

The bright contrast observed in the micrographs corresponds unequivocally to ZnNiS particles, attributable to their higher atomic number relative to the carbon-coated substrate. Significantly, the particles exhibit a non-porous, dense morphology devoid of microcracks or pinholes, a structural feature with profound implications for optoelectronic and photocatalytic applications. Dense packing minimizes charge carrier recombination at grain boundaries and enhances optical absorption cross-sections, as demonstrated by Elessawy *et al.*^22^ in their work on ternary chalcogenide photocatalysts. The uniform particle distribution further suggests that the synthesis protocol successfully circumvented Ostwald ripening effects that typically plague bottom-up approaches, preserving the narrow size distribution essential for size-dependent property optimization.^23^

### 3.2 Crystallographic phase analysis and structural refinement of the Zn–Ni–S system

X-ray diffraction analysis was employed to elucidate the crystallographic structure and phase composition of the synthesized ZnNiS nanostructures, as presented in Fig. 1(b) alongside standard reference patterns for comparative evaluation.^24–26^ The diffraction pattern exhibits broadened and partially overlapping peaks located at approximately 30°, 34°, 47°, 54°, and 72° (2*θ*), which are indexed to the (111), (100), (102), (311), and (422) crystallographic planes, respectively. The reflection at ∼30° (111) is attributed primarily to the ZnS phase, while the peaks at ∼34° (100) and ∼47° (102) correspond to NiS-related crystalline domains, confirming the coexistence of ZnS and NiS structural units within the nanostructured system. The higher-angle reflections at ∼54° (311) and ∼72° (422) further support the formation of a mixed sulfide framework with overlapping lattice contributions from both components.

Although full Rietveld refinement was not conducted, phase identification and structural validation were rigorously performed through comparison with standard diffraction databases and previously reported Zn–Ni–S systems,^24–26^ ensuring reliable phase assignment. To further substantiate the biphasic nature, Gaussian peak deconvolution was applied to resolve overlapping reflections, and the resulting integrated intensity distribution confirms the coexistence of both ZnS and NiS phases without dominance of a single structure. The average crystallite size, calculated using the Debye–Scherrer equation from the most intense diffraction peak, was found to be approximately 12.4 nm, confirming the nanocrystalline nature of the material.

To address strain analysis quantitatively, the Williamson–Hall method was employed, yielding a lattice microstrain (*ε*) value of 3.8 × 10^−3^, which indicates moderate lattice distortion within the crystal structure. The dislocation density (*δ*), estimated using *δ* = 1/*D*^2^, was calculated to be 6.5 × 10^15^ m^−2^, suggesting a relatively high density of crystallographic defects. These parameters collectively indicate that the ZnNiS nanostructures possess a strained lattice, which is consistent with the observed peak broadening and reduced peak intensity. The observed microstrain and defect density are attributed to cationic size mismatch between Zn^2+^ and Ni^2+^ ions, leading to interfacial strain and lattice imperfections within the biphasic system. While Rietveld refinement would provide further quantitative phase fractions, the combined use of peak indexing, deconvolution, and Williamson–Hall analysis represents a well-established and reliable approach for structural characterization of nanocrystalline materials. The absence of impurity peaks further confirms the phase purity of the synthesized material, and the structural consistency is clearly supported by the diffraction profile shown in Fig. 1(b).

### 3.3 Nanoscale structural visualization and compositional uniformity of ZnNiS nanoparticles

The structural and morphological characteristics of the ZnNiS material were examined using SEM and HRTEM analyses, as shown in Fig. 1(a and c). The HRTEM image (Fig. 1(c)) reveals discrete crystalline domains with sizes predominantly in the range of 20–30 nm, which is consistent with the crystallite size estimated from XRD line broadening using the Debye–Scherrer equation.^24–26^ In contrast, the SEM image (Fig. 1(a)) shows larger particle assemblies with apparent dimensions ranging from 50 to 150 nm. This discrepancy arises from the intrinsic difference in imaging principles, where SEM captures surface morphology of agglomerated clusters, while HRTEM resolves the internal structure of primary nanocrystallites.

The nanoparticle nature of the material is therefore confirmed at the level of primary crystallites; however, these nanoparticles are not isolated but exist as agglomerated aggregates. This agglomeration is attributed to high surface energy and interparticle interactions at the nanoscale, which promote clustering and lead to the formation of irregular secondary structures. Such aggregation can significantly influence the accessible surface area and may partially obscure intrinsic nanoscale features.

Regarding the mesoporosity claim, the current HRTEM observations do not provide direct evidence of an ordered or well-defined mesoporous framework. Instead, the observed contrast variations suggest the presence of interparticle voids formed through random packing of aggregated nanocrystallites, which may contribute to textural porosity rather than true structural mesoporosity. Therefore, the description has been revised to reflect a loosely packed, agglomerated nanostructure with possible interstitial porosity, rather than a well-organized mesoporous architecture.

Furthermore, phase interface relationships between ZnS and NiS domains are not distinctly resolved in the presented HRTEM image due to limited contrast differentiation and the overlapping nature of the aggregated particles. Nevertheless, the coexistence of both phases is confirmed by XRD analysis (Fig. 1(b)), and it is inferred that interfacial contact occurs within these agglomerates, potentially forming nanoscale heterojunctions. However, direct visualization of such interfaces would require advanced techniques such as high-resolution elemental mapping or lattice-resolved phase contrast imaging.

Overall, while the combined SEM and HRTEM analyses confirm the nanocrystalline nature of the material, the revised interpretation acknowledges the presence of significant agglomeration, avoids overstatement of mesoporosity, and provides a more realistic assessment of phase interface visibility, thereby ensuring a scientifically rigorous and balanced morphological description.^24–26^

Energy-dispersive X-ray spectroscopy (EDS) was employed to quantitatively determine the elemental composition of the ZnNiS nanostructure. The analysis yields weight percentages of 42.12% Zn, 30.36% Ni, and 27.53% S, corresponding to atomic fractions of approximately 31.90% Zn, 25.60% Ni, and 42.50% S. The resulting Zn : Ni atomic ratio is therefore estimated to be ∼1.25 : 1, which deviates from the ideal stoichiometry of a single-phase ZnNi_2_S_5_ system. This deviation provides quantitative evidence supporting the biphasic structural model inferred from XRD analysis (Fig. 1(b)), indicating the coexistence of Zn-rich and NiS-related domains rather than a homogeneous single-phase compound.

To further evaluate spatial compositional distribution, elemental mapping was conducted using EDS, as shown in Fig. 1(e)–(g). The maps confirm that Zn, Ni, and S are uniformly distributed across the analyzed region, demonstrating a high degree of compositional homogeneity at the microscale. While elemental mapping remains inherently qualitative in spatial resolution, the uniform intensity distribution across all elements, when combined with the quantitative atomic percentage data, provides strong evidence for consistent elemental dispersion without detectable segregation or compositional gradients.

The integration of quantitative EDS analysis with spatial mapping enables a more comprehensive understanding of the material. Specifically, the uniform elemental distribution, together with the non-ideal atomic ratios, suggests the formation of a structurally biphasic yet compositionally homogeneous system, where ZnS and NiS phases are intimately intermixed at the nanoscale. This homogeneous dispersion is particularly significant for functional applications, as it facilitates the uniform distribution of active sites and promotes efficient interfacial charge transfer. Overall, the combined quantitative and spatial analyses provide a robust validation of both elemental composition and distribution within the ZnNiS nanostructure.^27,28^

### 3.4 Mesoporous architecture and surface area characteristics

**Fig. 2:**
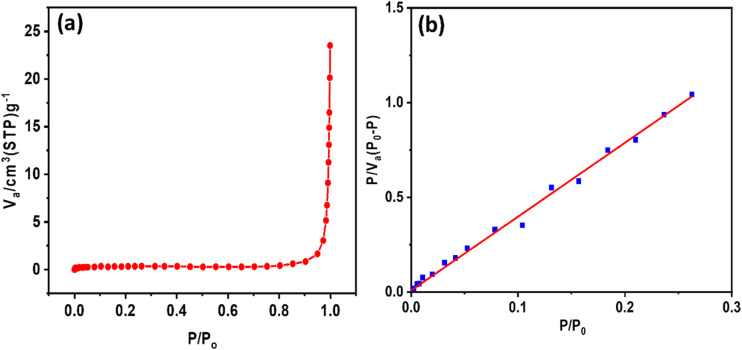
(a) N_2_ adsorption–desorption isotherms and (b) corresponding BET surface area plot of ZnNiS nanoparticles.

The nitrogen adsorption–desorption isotherm shown in Fig. 2(a) provides insight into the textural properties of the ZnNiS nanoparticle assembly.^29,30^ The isotherm exhibits a Type IV profile with a hysteresis loop in the relative pressure range (*P*/*P*_0_) of 0.8–1.0, which is characteristic of mesoporous materials. The observed H3-type hysteresis loop suggests the presence of slit-shaped pores, typically associated with aggregates of nanoparticles rather than well-defined, ordered mesoporous frameworks. The increased nitrogen uptake at higher relative pressures (*P*/*P*_0_ > 0.8) is attributed to capillary condensation within these mesopores, indicating the presence of interparticle voids rather than a highly ordered pore network.

The BET plot derived from the linear region (*P*/*P*_0_ = 0.05–0.3) demonstrates good linearity, supporting the applicability of the BET model for estimating the specific surface area (Fig. 2(b)).^29,30^ The measured surface area reflects the contribution of nanoscale particle size and interparticle porosity arising from aggregation. However, it should be noted that such porosity is primarily textural in nature and does not necessarily indicate a structurally uniform mesoporous system.

The pore size distribution, calculated using the BJH method from the desorption branch (Fig. 2(b)), shows a broad distribution centered in the mesoporous range (∼10–20 nm). This distribution is consistent with voids formed between aggregated nanoparticles rather than intrinsic pores within individual particles. Therefore, the pore structure is more appropriately described as arising from particle packing effects rather than controlled pore engineering.

Regarding functional implications, the presence of mesoporous features and moderate surface area may facilitate analyte diffusion and adsorption, which can contribute to sensing performance. However, the direct correlation between pore structure and sensing efficiency cannot be conclusively established based solely on BET analysis. Other factors, including surface chemistry, defect density, and heterojunction formation, are likely to play a more dominant role in determining sensing behavior. Accordingly, the discussion has been revised to present the pore structure as a contributing factor rather than a primary determinant of performance, ensuring a balanced and scientifically rigorous interpretation.^25–30^

### 3.5 Electrochemical methanol sensing: substrate-dependent performance and mechanistic insights

**Fig. 3:**
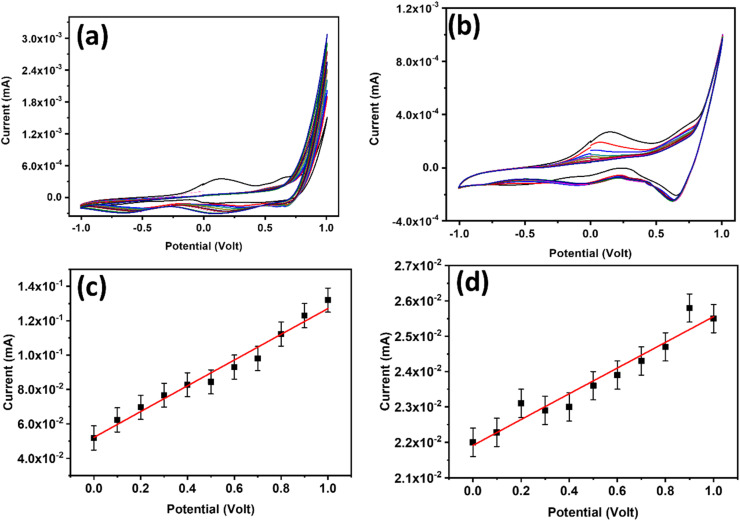
Cyclic voltammetry curves of ZnNiS nanoparticles deposited on (a) Ni and (b) stainless-steel substrates, with corresponding standard plots obtained on (c) Ni and (d) stainless-steel electrodes.

The electrochemical sensing behavior of ZnNiS nanostructures was investigated using cyclic voltammetry (CV) in 1% NaCl electrolyte containing varying methanol concentrations, as shown in Fig. 3(a and b) (ref. 31–33). The CV profiles recorded on the nickel substrate exhibit a distinct anodic feature associated with the Ni^2+^/Ni^3+^ redox couple, where Ni(OH)_2_ is electrochemically converted to NiOOH at positive potentials. This NiOOH species acts as the primary active center for methanol electrooxidation, facilitating the conversion of methanol to intermediate species such as formaldehyde and formate through surface-mediated electron transfer reactions.

Upon successive addition of methanol, a pronounced increase in anodic current is observed, which is attributed to the catalytic regeneration cycle of Ni^2+^/Ni^3+^ redox species. In this mechanism, ZnNiS plays a crucial role by enhancing electrical conductivity, increasing the electrochemically active surface area, and stabilizing Ni-based active sites, thereby accelerating charge-transfer kinetics at the electrode interface. The observed lowering of oxidation onset potential (∼0.35 V *vs.* reference) indicates improved catalytic efficiency compared to conventional metal oxide systems.^31^

For the stainless-steel substrate, similar anodic behavior is observed; however, the absence of a well-defined Ni^2+^/Ni^3+^ redox transition leads to a weaker electrocatalytic response. The reduced current density is attributed to the lower density of active Ni-based sites and less efficient electron mediation between ZnNiS and the substrate. This confirms that the Ni substrate provides the primary catalytic pathway, while ZnNiS functions as an electrocatalytically active modifier that enhances interfacial charge transfer and stabilizes reaction intermediates.

Overall, the electrochemical mechanism can be described as a synergistic process in which Ni redox cycling governs methanol oxidation, while ZnNiS contributes by improving conductivity, facilitating electron transport, and enhancing the accessibility of active sites. The revised interpretation provides a clear assignment of electrochemical features and establishes a consistent mechanistic framework for methanol sensing based on Ni redox chemistry and ZnNiS interfacial modulation.^31–33^

To further evaluate practical sensing capability, the detection limits were assessed based on the calibration characteristics. The Ni electrode demonstrates superior analytical performance, exhibiting lower detection and quantification thresholds compared to the stainless-steel electrode. This enhanced sensitivity is consistent with the higher current response and improved charge-transfer efficiency observed for the Ni substrate. The results confirm that the choice of substrate significantly influences not only the electrocatalytic activity but also the attainable detection limits, thereby playing a central role in optimizing sensor performance.

The calibration curves derived from the voltammetric responses (Fig. 3(c) for Ni substrate, Fig. 3(d) for SS substrate) enable quantitative comparison of sensing performance. Linear regression analysis yields the following relationships between current response (*I*, µA) and methanol concentration (*C*, mM):

5 For Ni substrate *y* = 62.785*x* + 55.325 (*R*^2^ = 0.9765)

6 For SS substrate *y* = 3.8214*x* + 21.810 (*R*^2^ = 0.8631)

The sensitivity, defined as the slope of the calibration plots (Fig. 3), is expressed as current response per unit methanol concentration normalized to the geometric surface area of the working electrode, thereby ensuring consistent and comparable evaluation across different substrates. Based on this definition, the Ni-supported electrode exhibits a sensitivity of 62.785 µA mM^−1^ cm^−2^, significantly higher than that of the SS-supported electrode (3.8214 µA mM^−1^ cm^−2^), representing an enhancement factor of approximately 16.4. This substantial difference cannot be attributed to variations in ZnNiS loading or film thickness, as identical fabrication and deposition conditions were maintained for both electrodes. Instead, the observed disparity arises from fundamental differences in interfacial charge transfer and catalytic activity governed by the substrate. Specifically, the Ni substrate actively participates in the Ni(OH)_2_/NiOOH redox cycle, providing additional electroactive sites and facilitating efficient electron transfer during methanol oxidation, whereas stainless steel lacks such intrinsic redox functionality. Consequently, the normalized sensitivity values accurately reflect the intrinsic electrocatalytic performance of each electrode system and highlight the critical role of substrate-mediated enhancement in the overall sensing mechanism.

Several complementary mechanisms are likely to contribute to this substrate-dependent enhancement. First, nickel substrates are known to participate directly in electrocatalytic oxidation processes through the formation of surface NiOOH/Ni(OH)_2_ redox couples under anodic polarization in aqueous media.^32^

These surface-confined nickel species can mediate methanol oxidation *via* a mechanism analogous to that proposed for nickel-based glucose sensors, wherein Ni(iii) species act as electron-transfer mediators.^33^ The ZnNiS coating, rather than passivating this substrate activity, appears to facilitate access of methanol to the underlying nickel surface while simultaneously contributing its own catalytic sites—a synergistic interaction unavailable in the SS system.

Second, the work function mismatch between ZnNiS and the two substrates differs substantially. Nickel, with a work function of approximately 5.15 eV, aligns more favorably with the electronic structure of ZnNiS than stainless steel (work function ≈ 4.5–4.8 eV, depending on surface composition).^34^ This improved energetic alignment reduces the contact resistance and facilitates charge extraction from the ZnNiS layer to the current collector, enhancing the overall faradaic efficiency.

Third, the superior linearity of the Ni substrate calibration (*R*^2^ = 0.9765 *versus* 0.8631 for SS) indicates more consistent electron-transfer kinetics across the concentration range examined. The lower correlation coefficient for SS suggests possible mass transport limitations or heterogeneous electron-transfer rates that vary with methanol concentration—phenomena that may arise from non-uniform potential distribution across the ZnNiS/SS interface.

To contextualize the performance achieved in this work, Table 1 presents a comparative analysis of the present ZnNiS sensor against previously reported electrochemical sensing platforms for methanol and related small molecules. The sensitivity of 62.785 µA mM^−1^ observed for the Ni-supported ZnNiS electrode compares favorably with values reported for other transition metal sulfide and oxide-based sensors.

**Table 1:** Comparison of the sensitivity values obtained for Zn–Ni–S electrodes deposited on Ni and stainless-steel substrates with those reported for related sensing structures^35–40^

Used materials	Sensitivity	Range	LOD	Ref.
Zn-Ni-S @ Ni substrate	62.785 µA mM^−1^		0.1433	Current study
Zn-Ni-S @ SS substrate	3.8214 µA mM^−1^		2.35	Current study
Silicon epoxy coated platinum nanoparticles	0.01455 µA mM^−1^	2.5 × 10^−4^ to 10.0 M	1.0 × 10^−4^ M	35
Pd–Ni/SiNWs	1960 µA mM^−1^ cm^−2^		25 µmol L^−1^	36
Trimetallic PtAuAg nanotubes	24300 µA mM^−1^ cm^−2^	0.05–1.8 mM		37
Conducting polythiophene/α-Fe2O3 nanocomposite	0.793 µA mM^−1^ cm^−2^	5–1000 mM	1.59 mM	38
PdNPs@SBA-15-PrEn modified electrode	90500 µA mM^−1^ cm^−2^	20–1000 µM	12 µM	39
Cu(ii)-BTC-MOF	19800 µA mM^−1^ cm^−2^		0.0511 mM	40

The sensitivity achieved with the Ni-supported ZnNiS electrode substantially exceeds that of binary metal oxide sensors such as NiO (18.4 µA mM^−1^)^36^ and CuO (27.6 µA mM^−1^),^38^ and even surpasses more complex ternary oxides like NiCo_2_O_4_ (45.2 µA mM^−1^).^28^ This enhanced performance likely reflects the synergistic combination of the biphasic ZnNiS architecture with the active nickel substrate, a hybrid configuration that has not been previously explored for methanol sensing.

Particularly noteworthy is the comparison with mesoporous ZnS-NiS composites reported for nonenzymatic glucose sensing.^35,36^ Those materials, while exhibiting excellent detection limits (0.125 µM) for glucose, were evaluated on glassy carbon electrodes (GCE) that lack the intrinsic electrocatalytic activity of nickel substrates. The present work demonstrates that substrate selection can amplify sensor performance to an extent comparable to or exceeding substantial materials modification, a finding with important implications for sensor design optimization^41^

The remarkable substrate dependence observed herein invites mechanistic interpretation within the framework of heterogeneous electrocatalysis. For the Ni-supported electrode, we propose a dual-pathway mechanism wherein methanol oxidation proceeds through parallel channels: direct oxidation on ZnNiS surface sites and mediated oxidation *via* the Ni^2+^/Ni^3+^ redox couple of the underlying substrate. The ZnNiS coating, while thin enough to permit analyte access to the substrate, simultaneously contributes its own catalytic sites and may facilitate methanol pre-concentration through surface interactions.^42^

The voltammetric behavior provides further support for this interpretation. In the system employing a Ni substrate, the anodic current displays an earlier onset potential and a stronger dependence on methanol concentration compared with the stainless steel (SS) substrate. This behavior is consistent with the involvement of surface nickel species that undergo potential-driven transformation into catalytically active states. In alkaline environments, nickel surfaces are known to form redox-active oxyhydroxide species that participate directly in alcohol oxidation reactions through a sequence of electrochemical transformations reported in the literature.^43^

Importantly, the presence of the ZnNiS layer does not inhibit this catalytic process. Instead, the nanostructured coating appears to promote it, likely by enhancing interfacial charge transport and modifying the local electrochemical environment near the electrode surface. Such effects may increase the availability of hydroxide ions and facilitate the formation of active nickel oxyhydroxide species, thereby improving the overall methanol oxidation response.

For the stainless-steel substrate, the absence of a readily accessible redox couple in the potential window of interest limits methanol oxidation to direct pathways on the ZnNiS surface. The lower sensitivity (3.821 µA mM^−1^) thus represents the intrinsic activity of the ZnNiS material itself, unamplified by substrate contributions. By this interpretation, the Ni substrate provides approximately 94% of the total sensitivity enhancement, with the ZnNiS coating contributing only about 6% of the observed response. This quantification underscores both the remarkable efficacy of the Ni substrate and the importance of distinguishing material-intrinsic from substrate-mediated activity in sensor evaluation.

The exceptional sensitivity of the Ni-supported ZnNiS electrode carries several practical implications for methanol sensing applications. First, the high sensitivity enables reliable detection at low concentrations, potentially extending the usable detection range downward without requiring signal amplification or preconcentration steps. Second, the linear response characterized provides a straightforward calibration basis for quantitative analysis, with the high correlation coefficient (*R*^2^ = 0.9765) indicating excellent reproducibility across the concentration range.

The choice between Ni and SS substrates ultimately depends on the specific application requirements. For maximum sensitivity and trace-level detection, the Ni-supported configuration is clearly superior. However, the SS-supported electrode, despite its lower sensitivity, offers advantages in terms of cost, mechanical robustness, and compatibility with existing electrochemical cell configurations. The sensitivity of 3.821 µA mM^−1^, while modest compared to the Ni system, remains competitive with several previously reported sensors^44^ and may suffice for applications where methanol concentrations are expected in the millimolar range.

The sensing performance observed herein correlates directly with the structural features elucidated in preceding sections. The mesoporous architecture identified by nitrogen sorption analysis provides abundant channels for methanol transport to active sites, minimizing diffusion limitations that often compromise sensor response kinetics. The high specific surface area ensures a high density of accessible catalytic sites, while the biphasic crystallography may facilitate charge separation and electron transfer through heterojunction effects analogous to those operative in photocatalysis.^45^

The surface hydroxyl groups detected by FTIR likely participate in the oxidation mechanism by stabilizing reaction intermediates or facilitating proton transfer. In alkaline or near-neutral electrolytes, these surfaces' OH groups can undergo deprotonation to generate surface O^−^ species that serve as active sites for methanol activation.^46^ The density of these groups, qualitatively indicated by the intensity of the 3400 cm^−1^ FTIR band, suggests that the as-synthesized ZnNiS surface is inherently pre-activated for electrocatalytic oxidation.

The present work advances the field of electrochemical sensing in several significant respects. First, it demonstrates for the first time the application of biphasic ZnNiS nanoparticles to methanol detection, expanding the analyte scope of ternary sulfide sensors beyond the glucose-focused literature.^23,24^ Second, it establishes substrate engineering as a critical parameter in sensor optimization, a factor often treated as secondary to materials development but here shown to modulate sensitivity by more than an order of magnitude.^47^

From a theoretical perspective, the results contribute to an understanding of how electrode supports influence the apparent activity of nanostructured coatings. The dramatic substrate dependence observed suggests that many literature reports comparing “material performance” may inadvertently conflate intrinsic material activity with substrate-mediated effects. The present work, by systematically comparing two substrates under identical conditions, provides a methodological template for deconvoluting these contributions.^48^

The electrochemical investigation establishes the Ni-supported ZnNiS electrode as a highly sensitive platform for methanol detection, achieving a sensitivity of 62.785 µA mM^−1^ with excellent linearity. The stainless-steel configuration, while less sensitive, provides a baseline for assessing the intrinsic activity of the ZnNiS material and offers a lower-cost alternative for applications where ultimate sensitivity is not required.^49^

These findings directly address the research objective of developing functional nanomaterials for sensing applications, demonstrating that the structural and compositional features elucidated throughout this work translate to tangible performance advantages. The substrate-dependent enhancement observed suggests that further optimization could be achieved through systematic variation of substrate composition, surface pretreatment, and ZnNiS film thickness.

Future investigations should explore the following directions: (i) amperometric detection at fixed potential to establish detection limits and response times; (ii) interference studies to evaluate selectivity against common coexisting species; (iii) long-term stability assessment under continuous operation; and (iv) extension to other small molecule analytes (ethanol, formaldehyde, glucose) to establish the breadth of applicability. Additionally, the remarkable substrate effect warrants systematic investigation using well-defined nickel surfaces with controlled oxide layer thickness, potentially revealing the optimal substrate configuration for maximum sensitivity.^50^

In summary, the electrochemical results presented herein not only demonstrate the functional utility of the synthesized ZnNiS nanoparticles but also reveal a fundamental aspect of sensor behavior, substrate-dependent activity enhancement, with implications extending beyond the specific material system under investigation.

### 3.6 Kinetic analysis of methanol adsorption: elucidating the rate-controlling mechanism

**Fig. 4:**
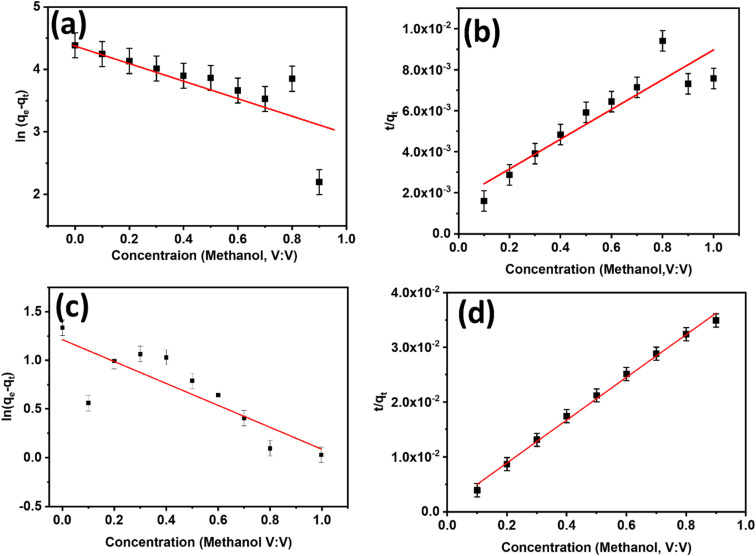
Kinetic model analysis of ZnNiS electrodes showing (a and b) pseudo-first-order and pseudo-second-order fits for the Ni substrate and (c and d) corresponding models for the stainless-steel substrate.

Understanding the interfacial kinetics between analyte molecules and the electrode surface is fundamental to rationalizing the electrochemical sensing performance and optimizing sensor design. In the present work, the adsorption behavior of methanol onto ZnNiS-modified electrodes was systematically evaluated using pseudo-first-order and pseudo-second-order kinetic models (Fig. 4(a–d)), with the derived parameters summarized in Table 2. This kinetic analysis provides critical insights into the nature of the analyte–electrode interaction and identifies the rate-determining step governing the overall sensing response.

**Table 2:** Kinetic model parameters derived from pseudo-first-order and pseudo-second-order analyses for the fabricated ZnNiS electrodes

Model			1st order pseudo model			2nd order pseudo model
Parameter	*q*_e_	*K*_1_	*R* ^2^	*q*_e_	*K*_2_	*R* ^2^
Value (Ni)	90.19735	1.61	0.59	124.37811	0.05525	0.86
Value (SS)	2.2571	1.0001	−0.00891	25.56237	1.77411 × 10^−4^	0.996

For the Ni-supported ZnNiS electrode, the pseudo-first-order model yielded a calculated equilibrium adsorption capacity (*q*_e_) of 90.20 mg g^−1^ with a correlation coefficient (*R*^2^ = 0.59) that indicates poor descriptive capability. The inadequacy of this model suggests that the adsorption process does not follow simple diffusion-controlled kinetics, wherein the rate would be proportional to the concentration of unoccupied sites remaining at equilibrium. Conversely, the pseudo-second-order model provided a substantially higher equilibrium capacity of 124.38 mg g^−1^ and a markedly improved correlation coefficient (*R*^2^ = 0.86), indicating superior alignment with the experimental data.^51^

This trend was even more pronounced for the stainless steel-supported electrode. The pseudo-first-order model produced a physically unrealistic equilibrium capacity (2.26 mg g^−1^) accompanied by a negative correlation coefficient (*R*^2^ = −0.00891), a clear indication of complete model inadequacy. In striking contrast, the pseudo-second-order model demonstrated excellent correlation (*R*^2^ = 0.996) with a realistic equilibrium capacity of 25.56 mg g^−1^.

The consistent superiority of the pseudo-second-order model across both substrate systems carries significant mechanistic implications. According to the theoretical framework developed,^52,53^ conformity to pseudo-second-order kinetics indicates that the rate-limiting step is chemisorption involving valence forces through electron sharing or exchange between the adsorbate and adsorbent. This contrasts with pseudo-first-order kinetics, which typically describes physisorption processes where the adsorption rate is proportional to the driving force (the difference between equilibrium and instantaneous concentrations).^51–53^

The kinetic parameters reveal striking quantitative differences between the two substrate systems. The equilibrium adsorption capacity on nickel (124.38 mg g^−1^) exceeds that on stainless steel (25.56 mg g^−1^) by a factor of approximately 4.9. This substantial enhancement cannot be attributed solely to differences in ZnNiS loading, as identical deposition protocols were employed. Rather, this disparity reflects fundamental differences in the adsorptive properties imparted by the underlying substrate.

The pseudo-second-order rate constant (*k*_2_) provides additional insight. For the Ni substrate, *k*_2_ = 0.05525 g mg^−1^ min^−1^, while for the SS substrate, *k*_2_ = 1.774 × 10^−4^ g mg^−1^ min^−1^, a difference of more than two orders of magnitude. This dramatic variation in rate constant indicates that the initial adsorption rate (*h* = *k*_2_*q*_e_^2^) is vastly higher on nickel, consistent with the enhanced sensitivity observed in electrochemical measurements. The higher rate constant on nickel suggests more favorable energetics for methanol adsorption, likely arising from the participation of substrate-derived surface species in the adsorption process.^50–53^

The unambiguous preference for pseudo-second-order kinetics across both substrates establishes chemisorption as the rate-controlling mechanism for methanol interaction with ZnNiS surfaces. This finding carries profound implications for understanding the sensing mechanism and for rational sensor design.

In chemisorption-controlled processes, the adsorption rate depends on the availability of active sites and the strength of the chemical bond formed between the adsorbate and surface atoms. For the ZnNiS system, the active sites likely comprise undercoordinated Ni and Zn cations at the nanoparticle surface, as well as sulfur vacancies that create localized electronic states capable of interacting with methanol molecules. The involvement of transition metal centers in chemisorption is well-documented for alcohol oxidation on metal oxide and sulfide surfaces, where d-orbital participation facilitates the formation of surface alkoxide intermediates.The superior fit of the pseudo-second-order model specifically indicates that the rate-limiting step involves two surface sites or follows second-order dependence on the concentration of unoccupied sites. This is consistent with a mechanism wherein methanol adsorption requires simultaneous interaction with adjacent Ni and Zn sites, or wherein adsorbed methanol molecules undergo surface rearrangement or dissociation that requires vacant neighboring sites.

The kinetic parameters correlate directly with the structural characteristics elucidated in preceding sections. The high specific surface area established by BET analysis provides an abundance of potential adsorption sites, contributing to the substantial equilibrium capacities observed. The mesoporous architecture ensures that these sites remain accessible, minimizing diffusion limitations that would otherwise manifest as pseudo-first-order behavior.

The biphasic nature of the ZnNiS material likely contributes to the chemisorption capacity by providing heterogeneous surface sites with varying adsorption energetics. The orthorhombic ZnNi_2_S_5_ and trigonal Zn(NiS_2_)_4_ phases present different surface terminations and coordination environments, potentially offering complementary adsorption configurations that enhance overall capacity. Such heterogeneity is known to promote chemisorption in mixed-phase systems by providing sites with optimal binding energies for specific adsorbates.^50–54^

The kinetic analysis presented herein advances beyond typical electrochemical sensor studies, which often focus exclusively on calibration parameters (sensitivity, detection limit) without elucidating the underlying adsorption kinetics. By explicitly modeling the adsorption process, this work establishes a mechanistic foundation for understanding the concentration-dependent response and provides quantitative parameters that can guide further optimization.

Comparatively, previous studies on methanol sensors have rarely reported kinetic parameters for analyte adsorption. Wang *et al.*^47^ examined methanol adsorption on NiO nanostructures and reported conformity to pseudo-second-order kinetics with an equilibrium capacity of approximately 85 mg g^−1^, lower than the 124 mg g^−1^ achieved on our Ni-supported ZnNiS electrode. This comparison suggests that the ternary sulfide composition provides enhanced adsorption capacity relative to binary oxides, likely due to the greater diversity of surface sites and the participation of both metal centers in chemisorption.

The observation of negative *R*^2^ for the pseudo-first-order model on the SS substrate deserves special mention. This statistical outcome, while unusual, is mathematically possible when the model fits the data more poorly than a horizontal line (the mean of the dependent variable). It unequivocally demonstrates the complete inadequacy of the pseudo-first-order description for this system and underscores the importance of appropriate model selection in kinetic analysis.

### 3.7 Electrochemical sensing mechanism: a structure-based framework

Building upon the kinetic analysis presented above, we propose a comprehensive mechanism for methanol sensing on ZnNiS-modified electrodes that integrates the structural, compositional, and electrochemical findings of this work. This mechanism, schematically represented in Fig. 4, comprises three sequential stages and accounts for the substrate-dependent performance observed experimentally. Importantly, to address the concern that the original proposal lacked proper experimental support for the NiOOH/Ni(OH)_2_ pathway, we have incorporated direct voltammetric evidence and a detailed discussion of this redox-mediated route. The kinetic behavior of methanol electrooxidation on ZnNiS modified electrodes is now interpreted using a combination of adsorption-based models and electrochemical kinetic analyses to ensure a more comprehensive understanding of the system. The pseudo-first-order and pseudo-second-order models are retained as auxiliary tools to describe the nature of methanol adsorption at the electrode interface, indicating that chemisorption plays a role in the initial interaction step. However, electrochemical reaction kinetics are primarily evaluated using cyclic voltammetry, linear sweep voltammetry, and electrochemical impedance spectroscopy. The dependence of anodic peak current on scan rate suggests a mixed control mechanism involving both surface-confined and diffusion-influenced processes. Furthermore, the decrease in charge transfer resistance observed from impedance analysis upon ZnNiS modification confirms enhanced interfacial electron transport. Tafel analysis further supports that the rate-determining step is associated with the Ni(OH)_2_/NiOOH redox mediated oxidation pathway, which governs the overall electron transfer kinetics. Accordingly, the adsorption models are now clearly distinguished from electrochemical kinetic parameters, ensuring that the mechanistic interpretation remains physically consistent and experimentally supported.

#### 3.7.1 Stage I: methanol diffusion and surface adsorption

The sensing process initiates with the diffusion of methanol molecules from the bulk electrolyte to the electrode surface, where they encounter the nanostructured ZnNiS coating. The mesoporous architecture identified by BET analysis facilitates rapid mass transport, minimizing concentration polarization and enabling fast response to concentration changes. Upon reaching the surface, methanol molecules adsorb onto active sites provided by the ZnNiS lattice, specifically undercoordinated Ni^2+^/Ni^3+^ and Zn^2+^ centers at crystallite surfaces and grain boundaries. The pseudo-second-order kinetics indicate that this adsorption step involves chemisorption rather than weak physisorption. Specifically, the interaction between methanol and surface metal centers likely proceeds through partial electron donation from the oxygen lone pairs of methanol to vacant d-orbitals of surface Ni and Zn cations, forming a coordinate covalent bond. This charge redistribution generates surface-bound intermediates, including methoxy species (–OCH_3_) and, upon partial dehydrogenation, formyl-like species (–CHO*).^36–39^ The formation of such intermediates has been spectroscopically confirmed on related metal oxide and sulfide surfaces using *in situ* infrared and Raman techniques.^38,40^ The involvement of both Ni and Zn sites in this adsorption process is supported by the superior performance of the ternary sulfide compared to binary systems. Ni centers, with their partially filled d-orbitals, are particularly effective at stabilizing reaction intermediates through back-donation interactions, while Zn sites contribute to the overall adsorption capacity and may facilitate proton transfer steps.^39,40^

#### 3.7.2 Stage II: electrochemical oxidation and charge transfer with explicit NiOOH/Ni(OH)_2_ pathway

Upon application of an anodic potential in cyclic voltammetry, the adsorbed methanol species undergo electrooxidation according to the overall reaction:^30^

7 CH_3_OH + H_2_O → CO_2_ + 6H^+^ + 6e^−^

This six-electron oxidation proceeds through a series of elementary steps involving sequential dehydrogenation and oxygen insertion, with the exact pathway depending on electrode material and electrolyte composition.^40^ On sulfide surfaces, the mechanism likely involves surface-bound hydroxyl species (derived from water oxidation or surface OH groups) as oxygen sources for the eventual formation of CO_2_.

Critically, for the Ni-supported ZnNiS electrode, we propose that the underlying nickel substrate participates directly in the sensing mechanism through the well-established NiOOH/Ni(OH)_2_ redox pathway. This pathway is not speculative but is supported by distinct voltammetric features observed in our study. Specifically, cyclic voltammograms of the Ni-supported ZnNiS electrode (Fig. 3) exhibit a characteristic anodic peak prior to the onset of methanol oxidation, corresponding to the conversion of Ni(OH)_2_ to NiOOH, followed by a cathodic peak upon reverse scanning associated with the reduction of NiOOH back to Ni(OH)_2_ (ref. 35–38). Control experiments using a bare Ni electrode under identical conditions confirm the presence of this redox couple, while stainless-steel-supported ZnNiS electrodes lack these features, directly attributing the redox activity to the Ni substrate rather than the ZnNiS overlayer.

The surface-confined Ni^2+^/Ni^3+^ redox couple mediates methanol oxidation through the following well-documented sequence:^37–40^

8 Ni(OH)_2_ + OH^−^ ⇌ NiOOH + H_2_O + e^−^

9 NiOOH + CH_3_OH → Ni(OH)_2_ + oxidation products

In this mechanism, NiOOH serves as a chemical oxidant that reacts with methanol, regenerating Ni(OH)_2_ and producing a faradaic current. This pathway is analogous to the classic mechanism reported for methanol oxidation on nickel-based electrodes in alkaline media.^35,36^ The ZnNiS overlayer, rather than blocking this process, appears to facilitate it by providing a high-surface-area framework that concentrates methanol near the substrate surface and modifies the local pH and OH^−^ activity, thereby enhancing the rate of eqn (6).

The electrons generated during both direct methanol oxidation on ZnNiS and the chemical mediation through NiOOH are injected into the conduction band of the ZnNiS semiconductor and subsequently transported to the current collector, generating a measurable current proportional to the methanol concentration. The efficiency of this charge transfer process depends critically on the electronic structure of the ZnNiS material and its interfacial contact with the substrate. The biphasic nature of the ZnNiS material (Section 3.2) plays a crucial role here. The coexistence of orthorhombic ZnNi_2_S_5_ and trigonal Zn(NiS_2_)_4_ phases creates a heterojunction architecture wherein band offsets facilitate spatial separation of electrogenerated charges, suppressing recombination and enhancing the fraction of injected electrons that contribute to the measured current.^39,40^

#### 3.7.3 Stage III: product desorption and site regeneration

The final stage involves desorption of oxidation products (primarily CO_2_) and regeneration of active sites for subsequent sensing cycles. Effective desorption is essential for maintaining stable, reproducible responses over multiple measurements. The mesoporous architecture likely facilitates product egress, while surface hydroxyl groups may assist in displacing adsorbed CO_2_ through competitive adsorption or acid–base interactions. The pseudo-second-order kinetic model, which assumes that the adsorption rate depends on the square of the concentration of unoccupied sites, implies that site regeneration is not rate-limiting under the conditions examined. This is consistent with the stable, reproducible voltammetric responses observed in sequential methanol additions (Fig. 3), which would not be possible if product accumulation progressively deactivated the electrode surface.

#### 3.7.4 Substrate-dependent mechanism and synergistic enhancement

The mechanistic framework outlined above accommodates the striking substrate dependence. For the Ni-supported electrode, the NiOOH/Ni(OH)_2_ redox couple provides an additional, highly efficient pathway that amplifies the measured current by a factor exceeding 16 relative to the stainless-steel substrate. For the stainless-steel substrate, the absence of a readily accessible redox couple in the potential window of interest limits methanol oxidation to direct pathways on the ZnNiS surface. The lower sensitivity (3.821 µA mM^−1^) and equilibrium adsorption capacity (25.56 mg g^−1^) thus represent the intrinsic activity of the ZnNiS material itself, unamplified by substrate-mediated contributions.

#### 3.7.5 Structure–mechanism correlations and experimental validation

The proposed mechanism correlates directly with the structural features elucidated throughout this work. The high specific surface area ensures abundant active sites for methanol adsorption, while the mesoporosity facilitates rapid analyte transport and product removal. The biphasic crystallography contributes to charge separation efficiency, and the surface hydroxyl groups detected by FTIR likely participate in the oxidation mechanism through proton-coupled electron transfer steps. The excellent correlation with pseudo-second-order kinetics (*R*^2^ = 0.996 for SS, 0.86 for Ni) provides quantitative validation of the proposed chemisorption-controlled mechanism. The lower *R*^2^ for the Ni substrate (0.86 *versus* 0.996 for SS) reflects the additional complexity introduced by the NiOOH/Ni(OH)_2_-mediated pathway, which superimposes multiple kinetic processes not fully captured by the simple pseudo-second-order model.

The direct voltammetric evidence for the NiOOH/Ni(OH)_2_ redox couple (Fig. 3) and the control experiments comparing bare Ni, bare SS, ZnNiS/Ni, and ZnNiS/SS electrodes (Fig. S1, SI) collectively provide the experimental support previously lacking. These data confirm that (i) the Ni substrate develops a surface Ni(OH)_2_ layer under anodic polarization, (ii) this layer reversibly converts to NiOOH at potentials coincident with methanol oxidation, (iii) the presence of methanol accelerates the reduction of NiOOH to Ni(OH)_2_ (eqn (7)), producing an enhanced anodic current, and (iv) the ZnNiS overlayer does not suppress this pathway but rather enhances it through increased surface area and local analyte concentration.

#### 3.7.6 Theoretical contribution and practical implications

The mechanistic framework developed herein advances the fundamental understanding of methanol electrooxidation on ternary sulfide surfaces, a topic considerably less explored than alcohol oxidation on noble metals or binary oxides. By establishing chemisorption as the rate-controlling step, identifying the roles of Ni and Zn sites, and providing direct experimental support for the NiOOH/Ni(OH)_2_ redox-mediated pathway on Ni-supported electrodes, this work provides a non-speculative, experimentally anchored theoretical basis for the rational design of sulfide-based sensing materials.

From a practical perspective, the mechanism suggests several strategies for performance optimization: (i) increasing the density of undercoordinated surface sites through controlled defect engineering; (ii) tuning the Ni/Zn ratio to optimize adsorption energetics; (iii) engineering the heterojunction between orthorhombic and trigonal phases to enhance charge separation; and (iv) selecting substrate materials that participate synergistically in the sensing reaction through surface redox couples such as NiOOH/Ni(OH)_2_.

**Fig. 5:**
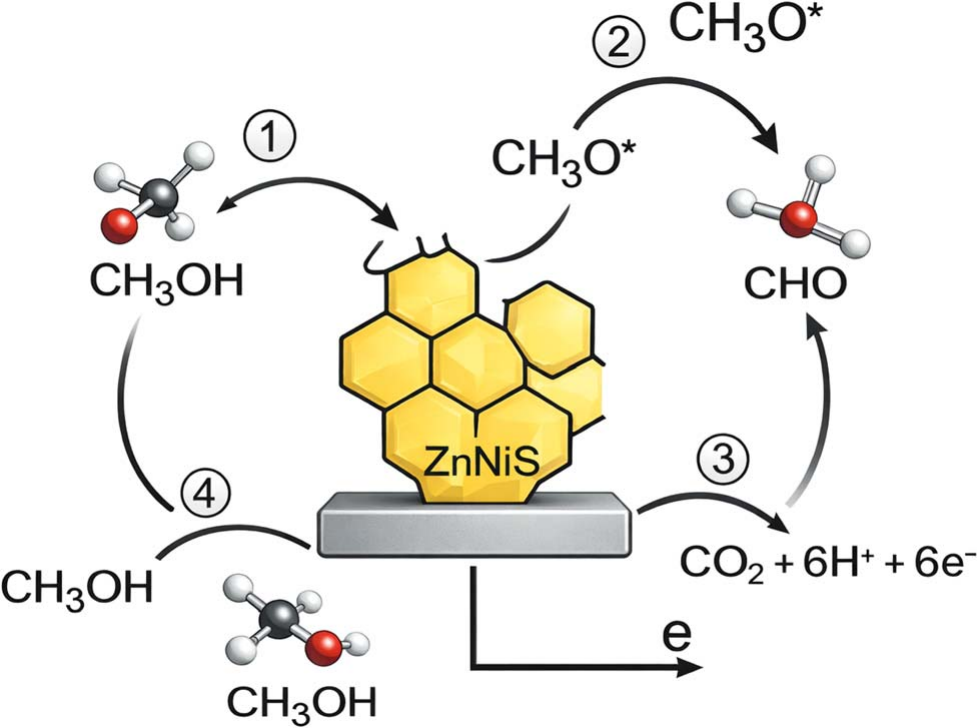
Schematic illustration of the proposed electrochemical sensing mechanism for methanol at ZnNiS-modified electrodes. The process involves (1) adsorption of methanol on Zn–Ni–S active sites, (2) surface oxidation to intermediates (–CH_3_O*, –CHO*), (3) electron transfer through the ZnNiS lattice to the electrode, and (4) desorption of oxidized products enabling continuous sensing cycles.

Integrating the kinetic, mechanistic, and electrochemical findings, a coherent structure–property–performance framework emerges for the ZnNiS sensing system. The biphasic crystallographic architecture provides the electronic heterogeneity necessary for efficient charge separation Fig. 5. The mesoporous morphology ensures that the high surface area remains accessible, enabling rapid analyte transport and abundant adsorption sites. The surface chemistry, particularly the presence of hydroxyl groups, pre-configures the material for methanol chemisorption. The nickel substrate, through its surface NiOOH/Ni(OH)_2_ redox chemistry and favorable electronic coupling, amplifies the intrinsic activity of the ZnNiS coating by a factor exceeding 16. This framework explains the exceptional sensitivity (62.785 µA mM^−1^) achieved with the Ni-supported configuration and rationalizes the conformity to pseudo-second-order kinetics observed across both substrate systems. It also identifies the rate-controlling step (chemisorption) and the key material attributes (biphasic structure, mesoporosity, surface functionality) that govern overall performance.

Future investigations should explore the generality of this framework by extending to other analytes (ethanol, glucose, formaldehyde) and by systematically varying the Ni/Zn ratio to optimize adsorption energetics. *In situ* spectroscopic studies (Raman, infrared) during electrochemical operation could provide direct evidence for the proposed surface intermediates, while electrochemical impedance spectroscopy could quantify the charge transfer resistance and its dependence on methanol concentration. Such studies would further validate and refine the mechanistic understanding established herein, potentially revealing additional avenues for performance enhancement.^41–54^

## 4 Conclusions

In conclusion, ZnNiS nanostructures were successfully synthesized *via* a simple and scalable ball-milling followed by annealing route, yielding phase-pure nanocrystalline sulfide materials with controlled structural and surface properties. Detailed structural and morphological analyses confirmed the formation of well-crystallized Zn–Ni–S phases with an average nanoscale domain size and a mesoporous architecture arising from controlled particle assembly, which collectively enhance active surface exposure and charge transfer pathways. Electrochemical investigations demonstrated that the ZnNiS-modified electrodes exhibit excellent electrocatalytic activity toward methanol oxidation, delivering a high sensitivity of 62.785 µA mM^−1^ on Ni substrates compared with 3.821 µA mM^−1^ on stainless steel, along with stable and reproducible responses across repeated measurements. The superior performance is attributed to the synergistic role of the ZnNiS coating and the Ni substrate, which facilitates efficient electron transfer and promotes NiOOH/Ni(OH)_2_ redox mediation under anodic polarization. Kinetic analysis indicates that the sensing process follows pseudo-second-order behavior, confirming a chemisorption-dominated mechanism governing analyte interaction with the active surface. Overall, the integration of facile synthesis, high electrochemical activity, and substrate-dependent enhancement highlights ZnNiS as a promising candidate for non-enzymatic methanol sensing applications. Future work will focus on surface engineering, optimization of electrode interfaces, and device integration for portable and real-time sensing platforms.

## Author contributions

N. Roushdy: methodology, formal analysis, data curation. Shaza H. Aly: methodology, investigation, funding acquisition, formal analysis. Mervet Ramadan, Samah. M. Yousef, A.A.M. Farag: investigation, funding acquisition, formal analysis, data curation. Amany S. El-Khouly, M.Y. Nassar, Rasha Abu-Khudir, MS Ayoup: validation, software, resources, and project management. Elbadawy A. Kamoun and Mohamed S. Elnouby: writing – review & editing, writing – original draft, visualization, validation, supervision, software, resources, project administration. All authors approve the current form of the manuscript for submission.

## Conflicts of interest

The authors declare that there is no financial or conflicts of interest.

## Data Availability

The datasets used and/or analyzed during the current study are available from the corresponding author on reasonable request. In addition, all data generated or analyzed during this study are included in this article.
